# The Story of the Insane from Year to Year

**Published:** 1904-05-28

**Authors:** 


### THE STORY OF THE INSANE FROM YEAR TO YEAR.

Sixteenth Annual Series.

### LONDON COUNTY ASYLUM AT CLAYBURY.

On the first of January, 1902, this asylum contained 2,406 patients, and on the last day of the year the number had fallen to 2,391. There were 373 first admissions, 72 re-admissions, 157 recoveries, 80 discharged relieved, and 223 died. The total number under treatment during the year was 2,851, and the average daily number resident was 2 398. Calculated on the admissions, the recoveries stand at 37·38 per cent., and calculated on the average number resident the deaths were 9 per cent., both of these being about the average of similar institutions. Of the year's deaths 55 were caused by cerebral and spinal diseases, 29 by pneumonia and broncho-pneumonia, 34 by consumption, 18 by dysentery and 1 by enteric. Of the year's admissions, 105 were supposed to owe their insanity to such moral causes as domestic trouble, adverse circumstances, mental anxiety, and religion while among the physical causes hereditary influence accounts for 114, congenital defect for 124, and intemperance in drink for 85. Dr. Jones seems to have taken much pains to ascertain the causes of insanity in those patients admitted to Claybury, and the "unknown" column is blank. As regards alcohol, it was the cause in 27 per cent. of the males and in 13 per cent. of the females, which he tells us is the highest percentage yet recorded at Claybury, and, high as it is, it is probably under the mark If to this 40 per cent. we add the 25 per cent. caused by heredity we arrive at this important fact—that 65 per cent. of the admissions had no right to exist at all; and we can only sigh for the advent of a higher civilisation which will declare that these things shall not be, and when that senseless cry, "the liberty of the subject," will not be applicable to those who think themselves quite free to make other people support their insane offspring. It is worthy of record that the Claybury Asylum has, since its opening, received more patients than has any other London asylum during the same period, and also that it has discharged more patients recovered than any other. It has been so far found impossible to eradicate asylum dysentery from Claybury. The drainage has been reconstructed, the cases are immediately isolated, those who recover are not warded with the other patients, and they are under strict observation for some time after their return to the ward. It is not easy to see what more could be done, and yet there were 91 cases. As regards consumption, there is not as yet any entirely separate accommodation for patients suffering from this disease, but separate wards are allotted to them, which is the next best thing that can be done. At Claybury much attention is given to systematic training of the attendants and nurses, and 36 obtained certificates in nursing during the year. The duration of the training is not specified. We hope it is a complete three years' course, and that those of the staff who obtain the higher certificate receive more wages. Dr. Jones must have gone through an anxious year. There have been many changes owing to illness among the higher staff, and it takes a while for new officers to settle themselves. Fortunately, Dr. Ewart remains on the staff, and Dr. Jones feelingly refers to that officer's long and valuable services, and to the interest he takes in his varied duties. The committee "acknowledge the manner in which their onerous duties have been performed by the medical superintendent and staff generally." The weekly cost per head is 12s. 2d.

